# Involvement of p53 in insulin-like growth factor binding protein-3 regulation in the breast cancer cell response to DNA damage

**DOI:** 10.18632/oncotarget.5612

**Published:** 2015-09-10

**Authors:** Kamila A. Marzec, Mike Z. Lin, Janet L. Martin, Robert C. Baxter

**Affiliations:** ^1^ Kolling Institute of Medical Research, University of Sydney, Royal North Shore Hospital, NSW, Australia

**Keywords:** insulin-like growth factor binding protein-3 (IGFBP-3), p53, DNA damage, breast cancer, chemotherapy

## Abstract

Chemotherapy drugs that induce apoptosis by causing DNA double-strand breaks, upregulate the tumor suppressor p53. This study investigated the regulation of the growth-regulatory protein insulin-like growth factor binding protein-3 (IGFBP-3), a p53 target, by DNA-damaging agents in breast cancer cells. IGFBP-3 was upregulated 1.4- to 13-fold in response to doxorubicin and etoposide in MCF-10A, Hs578T, MCF-7 and T47D cells, which express low to moderate basal levels of IGFBP-3. In contrast, IGFBP-3 was strongly downregulated by these agents in cells with high basal levels of IGFBP-3 (MDA-MB-231, MDA-MB-436 and MDA-MB-468). In MDA-MB-468 cells containing the R273H p53 mutation, reported to display gain-of-function properties, chemotherapy-induced suppression of IGFBP-3 was not reversed by the p53 reactivating drug, PRIMA-1, or by p53 silencing, suggesting that the decrease in IGFBP-3 following DNA damage is not a mutant p53 gain-of-function response. SiRNA-mediated downregulation of endogenous IGFBP-3 modestly attenuated doxorubicin-induced apoptosis in MDA-MB-468 and Hs578T cells. IGFBP-3 downregulation in some breast cancer cell lines in response to DNA-damaging chemotherapy may have clinical implications because suppression of IGFBP-3 may modulate the apoptotic response. These observations provide further evidence that endogenous IGFBP-3 plays a role in breast cancer cell responsiveness to DNA damaging therapy.

## INTRODUCTION

Breast cancer is the most common female cancer worldwide [1]. Although survival rates are high, many women still die from aggressive forms of this disease. Triple negative breast cancer (TNBC), which lacks estrogen and progesterone receptors (ER and PR), and HER2 amplification [2], which can be targeted by current therapies, presents a therapeutic challenge. Cytotoxic chemotherapy remains a front-line treatment in patients with TNBC, but is associated with severe side effects [3]. A majority, but not all, of TNBCs have a basal-like molecular profile [4]. A greater understanding of the molecular characteristics of TNBC may help to improve outcomes in patients with this disease.

Insulin-like growth factor binding protein-3 (IGFBP-3) is one of a family of six homologous proteins that bind IGF-1 and IGF-2 with high affinity, and have been implicated as both positive and negative regulators in many cancers [5]. IGFBP-3 contributes to cellular apoptosis in both IGF-dependent and -independent manners [6–12], although paradoxically, high IGFBP-3 mRNA and protein levels in breast tumors have been associated with aggressive forms of the disease [13–15], perhaps related to its ability to transactivate the EGF receptor [16], promote cell survival by autophagy [17], and contribute to the repair of DNA double-strand breaks [18]. Expression of IGFBP-3 is regulated by various factors, including the tumor suppressor protein, p53 [19, 20]. Consensus sites for p53 binding [21] have been identified in intronic regions of the *IGFBP3* gene [22], and wild-type p53 has been shown to upregulate IGFBP-3 following treatment with the DNA damaging agent doxorubicin in HeLa cervical cancer cells [22]. However, IGFBP-3 can also be upregulated in response to DNA damage in a p53-independent manner, as shown in p53-null PC-3 prostate cancer cells [23].

**Table 1:** Characteristics of the breast cell lines used in this study

Cell line	p53 status	Estrogen receptor status
MCF-10A	Wild type	Negative
MCF-7	Wild type	Positive
Hs578T	Mutant (V157F)	Negative
MDA-MB-231	Mutant (R280K)	Negative
MDA-MB-436	Mutant (E204fsX45)^a^	Negative
MDA-MB-468	Mutant (R273H)	Negative
T47D	Mutant (L194F)	Positive

a fs, frameshift; X, stop codon

Wild-type p53 regulates the transcription of many genes encoding proteins that mediate DNA repair, cell cycle control and apoptosis [24]. *TP53* is the second most frequently mutated gene in breast cancer (23%) after *PIK3CA* (26%) [25] and is considered among the key driving factors in TNBC - the most aggressive breast cancer subgroup [26]. Depending on the type of mutation, normal p53 function may be lost to varying degrees, allowing damaged cells to progress to a cancerous state. The most common p53 alterations are missense mutations of residues R175, Y220, G245, R248, R249, R273 and R282 in the DNA binding domain, referred to as “hotspots” [27]. Some mutations cause p53 to carry out functions that are opposite to those of wild type p53, allowing cancer cells to bypass apoptosis even in the presence of DNA damage, a phenomenon termed mutant p53 gain-of-function [28].

Since overexpressed and exogenous IGFBP-3 have been shown to contribute to apoptosis induced by DNA damaging agents [29–31], it is important to understand how such drugs affect endogenous IGFBP-3 expression. Wild type p53 stabilization, nuclear accumulation and activation are induced by similar stimuli to those that up-regulate IGFBP-3, including DNA damage or genotoxic stress, hypoxia and oncogene activity [20]. Since IGFBP-3 can act as a pro-apoptotic factor following DNA damage, even in the absence of p53 [8] or in the presence of mutant p53 (e.g. the L194F mutation in T47D cells) [29], it is possible that oncogenic forms of p53 might suppress IGFBP-3 and confer a survival advantage on a cancer cell under circumstances where IGFBP-3 is pro-apoptotic. Understanding the regulation of IGFBP-3 expression and actions when p53 is activated, such as during DNA damage, may contribute to a more comprehensive characterization of breast tumors and lead to more effective methods of treatment.

## RESULTS

### IGFBP-3 mRNA is expressed at different basal levels in various breast cell lines

**Figure 1:**
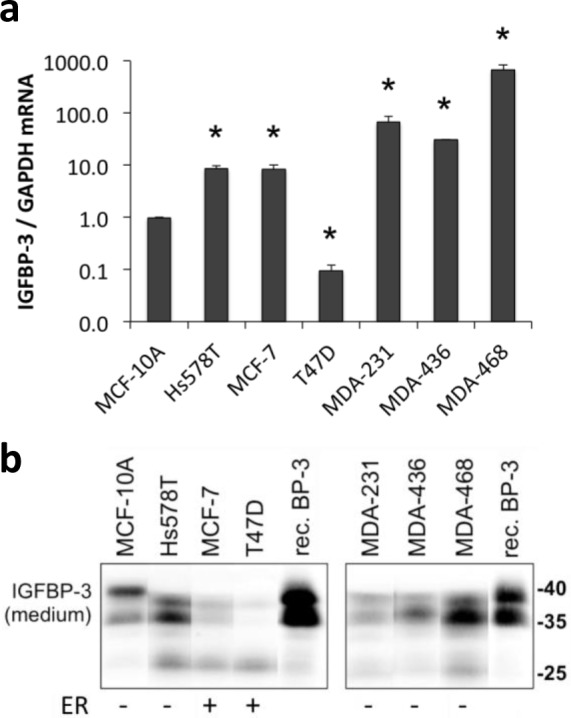
IGFBP-3 expression in breast cell lines Cells were plated at 2-3×10^5^ cells/well in 6-well plates before collecting medium and harvesting 48 h later. **a.** IGFBP-3 mRNA levels were quantified by qRT-PCR, normalized to GAPDH. Experiments were performed up to 3 times in duplicate for each comparison; data are mean values ± SEM. *P < 0.05 relative to MCF-10A. **b.** Western blots for IGFBP-3 in conditioned medium, using 5 ng recombinant IGFBP-3 (rec. BP-3) as a standard. Note the band at ~27 kDa representing proteolysed IGFBP-3 in some samples. Representative bands are from lanes on the same gel. Lanes between the shown lanes have been removed to create the presented image. ER, estrogen receptor positive or negative cell lines.

The expression of IGFBP-3 by breast cancer cells has been reported to correlate with ER status [32]. Relative amounts of IGFBP-3 mRNA and protein were compared in seven cell lines by plating cells at similar densities and harvesting after 24 h for analysis of IGFBP-3 mRNA by qRT-PCR, and IGFBP-3 protein secreted into media by immunoblotting. The ER-negative basal-like MDA-MB-468 cells expressed IGFBP-3 mRNA at the highest level (approximately 600-fold greater than the phenotypically normal breast epithelial cell line MCF-10A, which is also ER-negative). MDA-MB-231, MDA-MB-436 and Hs578T cells had 65-fold, 30-fold and 8-fold greater levels, respectively, of IGFBP-3 mRNA than MCF-10A cells (Figure 1a). The ER-positive cell line, MCF-7, also showed 8-fold higher IGFBP-3 mRNA levels than MCF-10A cells. T47D, another ER-positive cell line, had the lowest level of IGFBP-3 mRNA, 90% lower than MCF-10A cells. Therefore the basal levels of IGFBP-3 mRNA expression did not always correlate with ER status. In contrast, Western blot analysis showed that the levels of secreted IGFBP-3 in the conditioned medium, visible as a 35-40 kDa doublet, were highest in the ER-negative breast cancer cell lines compared with the ER-positive lines (Figure 1b).

### IGFBP-3 is downregulated in response to DNA damaging agents in breast cell lines that express high basal levels of IGFBP-3

**Figure 2:**
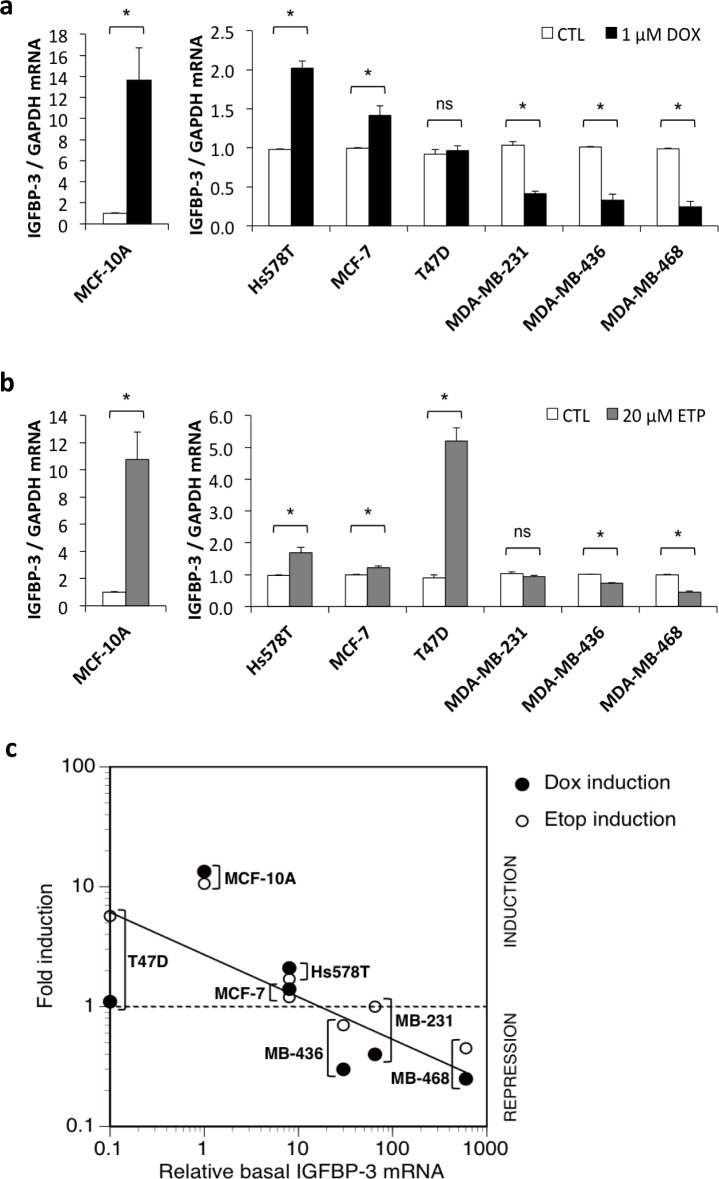
IGFBP-3 regulation by DNA-damaging drugs in breast cells Cells were plated at 2-3×10^5^ cells/well in 6-well plates. Cells were harvested and conditioned medium collected after 24 h treatment with **a.** 1 μM doxorubicin (DOX) or **b.** 20 μM etoposide (ETP). IGFBP-3 mRNA levels were measured by qRT-PCR, normalized to GAPDH. Treatment effects are shown relative to untreated control (CTL) for each cell line. Experiments were performed at least twice in duplicate for all cell lines. Data are mean values ± SEM; *P < 0.05; ns, not significant. **c.** The induction or repression of IGFBP-3 mRNA after doxorubicin or etoposide treatment is inversely related to basal IGFBP-3 mRNA levels.

In order to investigate whether different basal IGFBP-3 levels were associated with IGFBP-3 mRNA and protein regulation following DNA damage, cells were exposed to 1 μM doxorubicin or 20 μM etoposide for 24 h and the effects on IGFBP-3 expression were analysed. Drug doses and treatment times were based on optimization experiments where maximum apoptosis (discussed later) was observed. Following doxorubicin treatment, IGFBP-3 mRNA was increased significantly in MCF-10A, Hs578T and MCF-7 cells, by 13.4-fold, 2.1-fold and 1.4-fold, respectively (Figure 2a) and did not change significantly in T47D cells. By contrast, IGFBP-3 mRNA was significantly downregulated by doxorubicin in MDA-MB-231, MDA-MB-436 and MDA-MB-468 (Figure 2a). Etoposide treatment produced similar results with IGFBP-3 mRNA significantly increased in MCF-10A, Hs578T and MCF-7 cells, and a 5.7-fold increase in IGFBP-3 mRNA also apparent in T47D cells (Figure 2b). Etoposide significantly decreased IGFBP-3 mRNA in MDA-MB-436 and MDA-MB-468 cells, but was without effect in MDA-MB-231 cells under the conditions tested (Figure 2b). Following 24 h of treatment with either drug, the amount of secreted IGFBP-3 in the medium of Hs578T cells was unchanged (Supplementary Figure S1). Notably, secreted IGFBP-3 was decreased by approximately 33% in medium from MDA-MB-468 cells following doxorubicin treatment and by approximately 50% following etoposide treatment (Supplementary Figure S1).

These data show that in breast cell lines with the highest basal levels of IGFBP-3 mRNA, DNA damaging agents cause a decrease in its expression, whereas in cell lines in which basal expression is low, IGFBP-3 mRNA is increased by these drugs (Figure 2c). Collectively these results indicate a relationship between basal levels of IGFBP-3 mRNA and the regulation of IGFBP-3 in response to DNA damage in breast cells.

### Doxorubicin induces greater apoptosis in Hs578T cells where IGFBP-3 is upregulated compared to MDA-MB-468 cells where IGFBP-3 is downregulated

**Figure 3:**
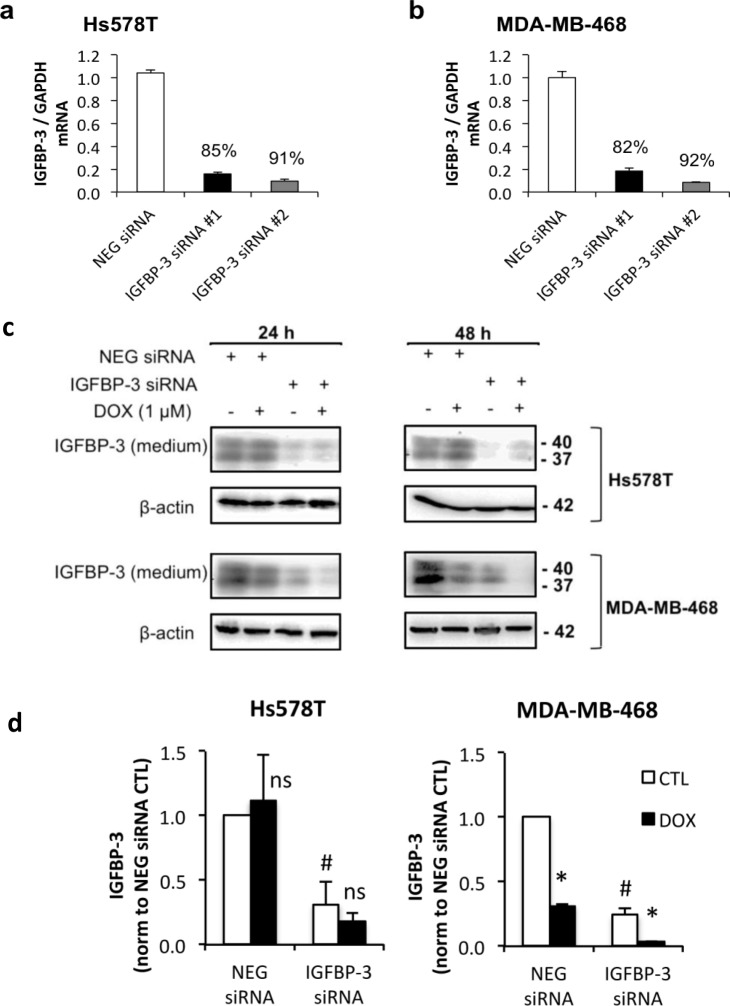
The effect of IGFBP-3 silencing on the response to doxorubicin in Hs578T and MDA-MB-468 cells Cells were harvested 24 h after transfection with negative control (NEG) siRNA or one of two IGFBP-3 siRNAs (#1 and #2). **a.** and **b.** Total RNA was normalized in the lysates before performing qRT-PCR to measure IGFBP-3 mRNA. GAPDH was the reference gene. Data represent mean values ± SEM of at least two experiments in duplicate for each cell line. The average percentage knockdown of IGFBP-3 by each siRNA is indicated. **c.** Western blot of IGFBP-3 in conditioned medium 24 h and 48 h after doxorubicin treatment following 24 h IGFBP-3 silencing. **d.** 48 h doxorubicin treatment quantified by densitometry. Mean values ± SEM; #*P* < 0.05 NEG siRNA *vs* IGFBP-3 siRNA (CTL only); *P < 0.05 CTL *vs* DOX; ns, not significant.

In order to test whether differential regulation of IGFBP-3 in response to DNA damage affects the cells’ response to these drugs, the apoptotic response to treatment with doxorubicin was measured by caspase-3 activation. Apoptosis in MDA-MB-468 cells, in which IGFBP-3 is downregulated by doxorubicin, was compared to that in Hs578T cells, where IGFBP-3 is upregulated. The involvement of IGFBP-3 in the apoptotic effects of doxorubicin in these lines was investigated by concomitant silencing of IGFBP-3 expression using either of two siRNA constructs targeting IGFBP-3, termed siRNA #1 and siRNA #2. IGFBP-3 mRNA was decreased by 85% and 91% in Hs578T cells (Figure 3a) and by 82% and 92% in MDA-MB-468 cells (Figure 3b) with siRNA #1 and siRNA #2, respectively. Secreted IGFBP-3 protein was also reduced by >50% in both cell lines 48 h after siRNA transfection, and showed little or no new production over the subsequent 24-48 h period (Supplementary Figure S2). In response to doxorubicin treatment, IGFBP-3 protein levels in IGFBP-3 siRNA cells followed the same pattern of regulation as negative control siRNA cells, as shown in Figure 3c and quantitated in Figure 3d.

**Figure 4:**
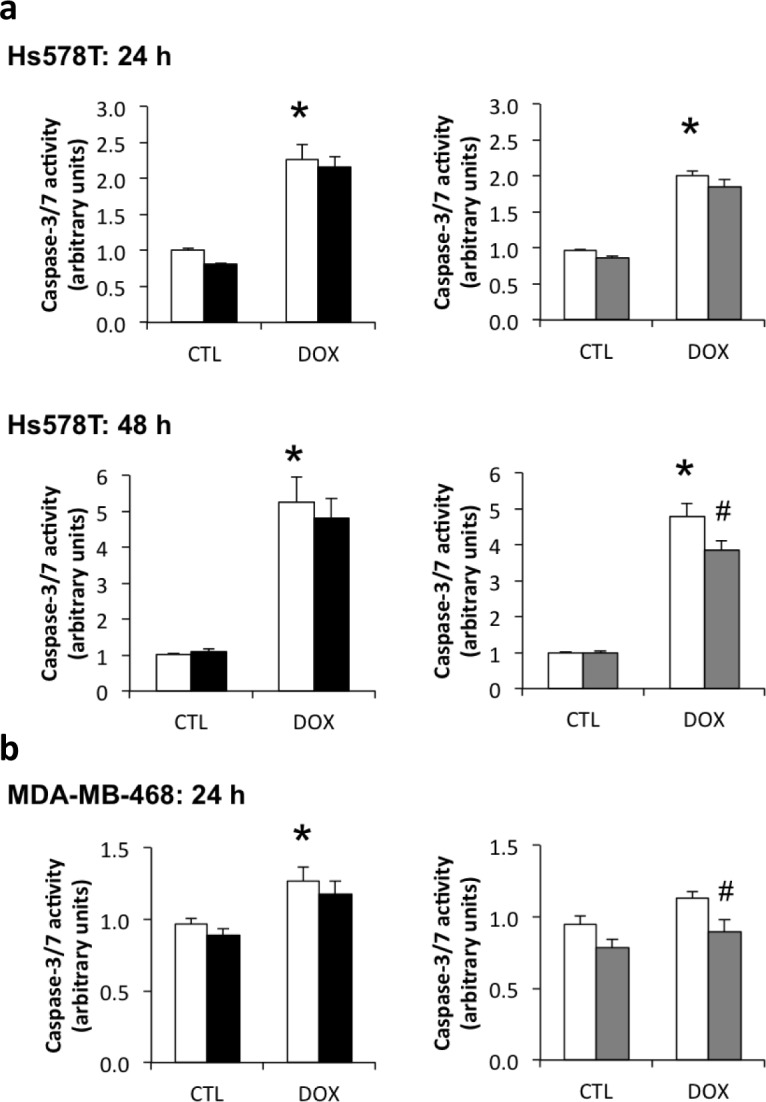
Contribution of endogenous IGFBP-3 to doxorubicin-induced apoptosis in Hs578T and MDA-MB-468 cells Cells were harvested 24 h after transfection with negative control (white bars) siRNA or one of two IGFBP-3 siRNAs (#1 and #2, black and gray bars, respectively). **a.** and **b.** Cells were treated with 1 μM doxorubicin (DOX) 24 h after siRNA transfection, and harvested 24 h and 48 h after DOX treatment. Total protein concentration was normalized in the lysates before fluorometric caspase-3/7 activation assay. Data are mean values ± SEM of at least three experiments in duplicate for each cell line and each treatment. *P < 0.05 NEG siRNA DOX *vs*. control untreated (CTL); #*P* < 0.05 IGFBP-3 siRNA *vs*. NEG siRNA.

Doxorubicin induced a 2-fold increase in apoptosis (measured as caspase3/7 enzyme activity) in Hs578T cells at 24 h of treatment and a 5-fold increase at 48 h (Figure 4a), while in the MDA-MB-468 cells, apoptosis was increased by only 20% (Figure 4b) at 24 h of treatment with no change at 48 h (data not shown). SiRNA-mediated knockdown of IGFBP-3 of >90% using siRNA #2 slightly decreased caspase-3/7 activation following treatment with doxorubicin compared to cells transfected with negative control siRNA. This was significant in Hs578T cells treated with doxorubicin for 48 h (Figure 4a), and MDA-MB-468 cells treated for 24 h (Figure 4b). Although a trend towards reduced caspase-3/7 activity was also apparent with siRNA #1 in both cell lines, the changes were not significant. These data suggest that endogenous IGFBP-3 makes a relatively small contribution to apoptosis induced by doxorubicin, measured at 24 or 48 h.

### No relationship between p53 status and IGFBP-3 regulation following DNA damage

The unexpected observation in MDA-MB-468 cells of DNA damaging agents downregulating IGFBP-3 mRNA and protein raised the possibility that the p53 mutation within these cells (R273H) may have a role in suppressing IGFBP-3 expression. MDA-MB-468, along with four other cell lines in this study (Hs578T [V157F], T47D [L194F], MDA-MB-231 [R280K] and MDA-MB-436 [E204Fs]), express mutant forms of p53, although only R273H is considered a “hotspot” mutation, with previously described gain-of-function properties [33–35]. The MCF-10A and MCF-7 cell lines express wild type p53 [36]. P53 expression was evaluated in all seven cell lines by Western blot, using an antibody that detects wild type and full-length mutant p53, following 4 h treatment with 1 μM doxorubicin or 20 μM etoposide. As previously reported in other cell types [37] total p53 protein levels were much higher in cells expressing mutant p53 compared to those expressing wild type p53 (Supplementary Figure S3). The exception to this was the MDA-MB-436 cell line, where p53 could not be detected with the antibody used due to the presence of a frameshift truncating mutation [38]. Although the immunoreactive p53 band was faint in MCF-10A and MCF-7 cells under basal conditions, doxorubicin and etoposide caused an increase in total p53 levels within 4 h of treatment. These drugs also increased total p53 in Hs578T cells, but had no effect over this time-course in the other cell lines. Doxorubicin and etoposide induced p53 activation in all cell lines, which was reflected in increased phosphorylation at serine-15 (Supplementary Figure S3). The level of phosphorylation varied in the different cell lines, being barely detectable in the wild type p53 cell lines MCF-10A and MCF-7. The results showed that p53 was easily detectable in mutant p53-containing cells, even under basal conditions, while p53-inducing conditions, such as exposure to DNA damaging agents, were required to detect it in wild type p53-containing cells. There was no clear relationship between the basal levels of p53, and either the basal expression of IGFBP-3 or whether it is up- or downregulated in response to doxorubicin or etoposide.

### PRIMA-1 induces PARP cleavage in MDA-MB-468 cells but not MCF-10A cells

**Figure 5:**
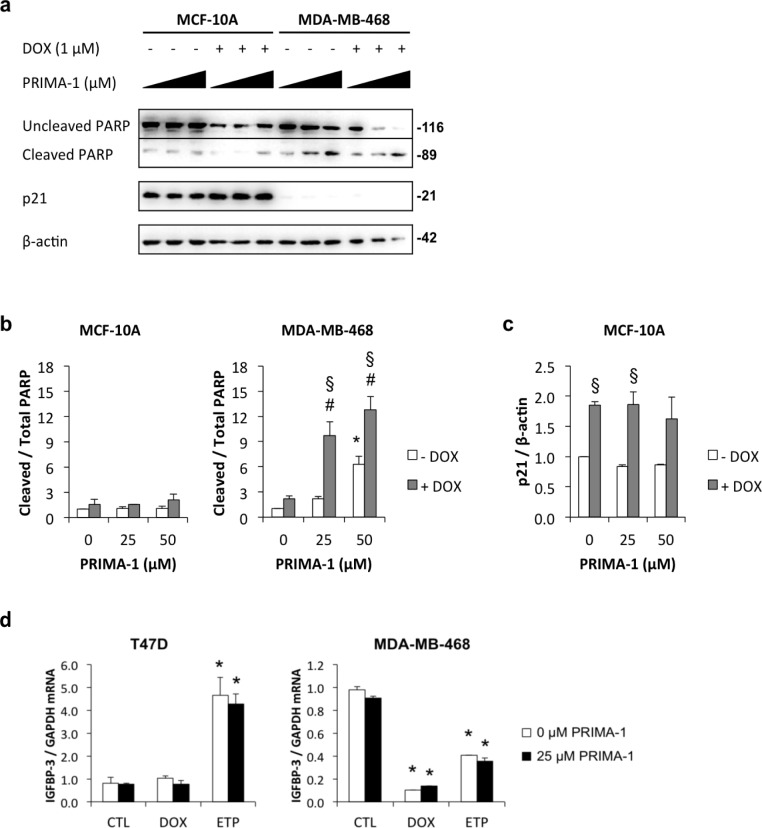
Induction of apoptosis by PRIMA-1 in MDA-MB-468 cells Cells were harvested 48 h after treatment with 1 μM doxorubicin (DOX) in the presence of increasing doses of PRIMA-1 (0, 25, or 50 μM). **a.** Lysates were analysed by Western blotting for uncleaved and cleaved PARP, p21, and β-actin. Signal intensities for **b.** uncleaved and cleaved PARP and **c.** p21 were measured by densitometry and normalized against β-actin. Data are mean values ± SD; *P < 0.05 PRIMA-1 effect (no DOX); #*P* < 0.05 PRIMA-1 (with DOX) vs PRIMA-1 (no DOX); §*P* < 0.05 DOX effect. **d.** T47D and MDA-MB-468 cells were treated with 1 μM DOX or 20 μM etoposide (ETP) in the absence and presence of 25 μM PRIMA-1. IGFBP-3 mRNA levels were measured by qRT-PCR using GAPDH as the reference gene. Data are mean values ± SD. *P < 0.05 DOX or ETP *vs* the corresponding CTL. The effect of PRIMA-1 was not significant.

The p53-reactivating drug, PRIMA-1 [39], was used to examine the role of p53 in downregulating IGFBP-3 in MDA-MB-468 cells. PRIMA-1 restoration of wild type p53 function was investigated by its ability to induce apoptosis and modulate p53-transcriptional targets in mutant p53-expressing cells. Using MDA-MB-468 cells, which have the R273H p53 mutation, and MCF-10A cells as a wild type p53-expressing control cell line, it was found that PRIMA-1 alone at 25 and 50 μM induced apoptosis, measured as cleavage of poly ADP-ribose polymerase (PARP), in the MDA-MB-468 cell line only (Figure 5a and 5b). It also significantly enhanced doxorubicin-induced PARP cleavage in the MDA-MB-468 cell line and not in the MCF-10A cell line. Figures 5a and 5c show that the p53 target p21 was significantly upregulated by doxorubicin treatment in MCF-10A cells but treatment with PRIMA-1 had no effect. In contrast, p21 protein was barely detectable in MDA-MB-468 control untreated cells and did not change in response to doxorubicin in the absence or presence of PRIMA-1 treatment (Figure 5a). These data suggest that PRIMA-1 selectively induces apoptosis in mutant p53-containing cells only, but is unable to induce p21, a transcriptional target of p53, even under p53-stimulating conditions such as DNA damage.

### PRIMA-1 does not reverse DNA damage-induced downregulation of IGFBP-3 mRNA in MDA-MB-468 breast cancer cells

Despite its ineffectiveness at inducing p21, a known transcriptional target of p53, PRIMA-1 was further investigated to assess its effect on IGFBP-3 regulation in MDA-MB-468 cells. PRIMA-1 (25 μM) had no effect on doxorubicin- or etoposide-induced regulation of IGFBP-3 mRNA in T47D and MDA-MB-468 cells (Figure 5d). Thus, as with its lack of effect on p21, PRIMA-1 had no effect on the DNA damage-induced downregulation of IGFBP-3 in MDA-MB-468 cells. PRIMA-1 may be considered an effective tool for enhancing DNA damage-induced apoptosis in mutant p53-expressing cells, but the mechanism by which it does so appears not to involve either p21 or IGFBP-3 regulation.

### IGFBP-3 downregulation in response to DNA damage is independent of mutant p53 in MDA-MB-468 cells

**Figure 6:**
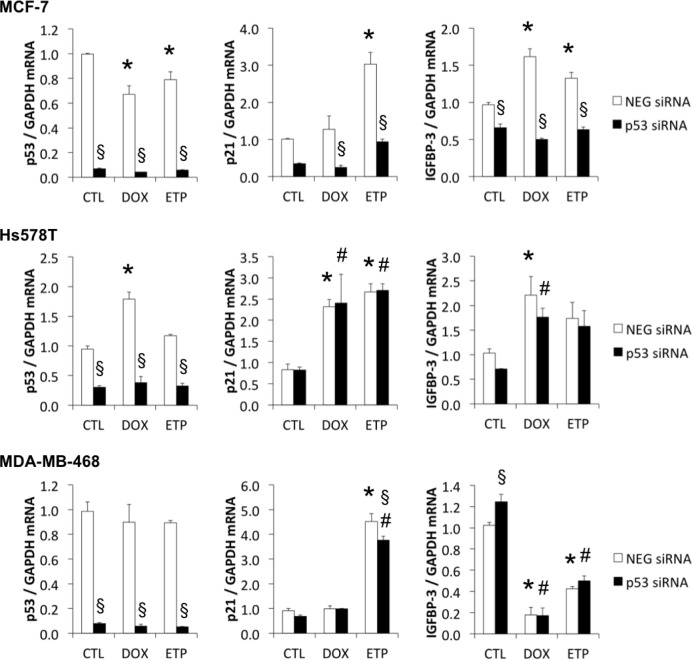
Mutant p53 has little effect on DNA damage-induced p21 and IGFBP-3 mRNA responses MCF-7, Hs578T and MDA-MB-468 cells were harvested 48 h after p53 siRNA transfection and following 24 h treatment with 1 μM doxorubicin (DOX) or 20 μM etoposide (ETP) (black bars). P53, p21 and IGFBP-3 mRNA levels were measured by qRT-PCR using GAPDH as the housekeeping gene. CTL, control cells (white bars); NEG, negative siRNA. Experiments were performed twice in duplicate for all cell lines; data represented as mean ± sem. *P < 0.05 NEG siRNA DOX/ETP compared to NEG siRNA CTL; #*P* < 0.05 p53 siRNA DOX/ETP compared to p53 siRNA CTL; §*P* < 0.05 p53 siRNA compared to NEG siRNA.

If IGFBP-3 downregulation by DNA damage is a mutant p53 gain-of-function, it should be abolished when the mutant p53 is downregulated. This was investigated using siRNA-mediated silencing of p53 in three cell lines - one expressing wild-type p53 (MCF-7) and two TNBC cell lines expressing mutant forms of p53 (V157F in Hs578T and R273H in MDA-MB-468) (Figure 6). Knockdown of p53 mRNA was between 83% and 95% in all cell lines, measured at the time of DNA-damaging treatment (0 h) (data not shown), and it remained strongly downregulated 48 h later (Figure 6). At 24 h following DNA damaging treatment, wild type p53 knockdown in MCF-7 cells resulted in a significant decrease in both doxorubicin- and etoposide-induced p21 and IGFBP-3 mRNA (Figure 6). By contrast, siRNA-mediated downregulation of mutant p53 in Hs578T cells had no effect on doxorubicin- or etoposide-induced p21 and IGFBP-3 mRNA levels. Similarly, despite a 92% silencing of mutant p53 mRNA in MDA-MB-468 cells, induction of IGFBP-3 mRNA by doxorubicin and etoposide was unaffected, although etoposide-induced p21 mRNA showed a small reduction as a result of p53 knockdown (Figure 6). Unexpectedly, IGFBP-3 mRNA in untreated MDA-MB-468 cells was significantly increased when p53 was silenced (Figure 6). Taken together, these results suggest that the mutant forms of p53 in Hs578T and MDA-MB-468 cells have little or no involvement in regulating IGFBP-3 mRNA following DNA damage.

The effect of silencing p53 on p21 and IGFBP-3 protein expression in these cell lines was confirmed by Western blot. As shown in Supplementary Figure S4a, wild type p53 was effectively knocked down by siRNA transfection in MCF-7 cells, as shown by a decrease in doxorubicin-induced p53 levels, and was associated with suppression of p21. P53 silencing in cell lines harboring mutant p53 resulted in significant reduction in p53 protein but had no effect on p21 expression in MDA-MB-468 and Hs578T cells (Supplementary Figure S4b). Finally, the knockdown of mutant p53 in Hs578T and MDA-MB-468 cells caused a small decrease in IGFBP-3 in the conditioned medium of these cells, significant only in etoposide-treated Hs578T cells (Supplementary Figure S5). Therefore, the data confirm at the protein level that mutant p53 plays little or no role in the downregulation of IGFBP-3 by doxorubicin and etoposide in MDA-MB-468 cells, suggesting that this effect, although opposite to the induction of IGFBP-3 by wild-type p53, is not a mutant p53 gain-of-function. As other p53 family members may have a role in IGFBP-3 regulation, we also tested a variety of cell lines for the presence of p63 and its truncated oncogenic form ΔNp63α [40] (Supplementary Figure S6). Only the TNBC cell line HCC1806 expressed detectable full-length p63 (at approximately 75 kDa by Western blot), and none had detectable ΔNp63α (at approximately 40 kDa). Therefore, despite a report that ΔNp63α can downregulate IGFBP-3 in squamous epithelial cell lines [41], this does not appear to be a likely mechanism of IGFBP-3 downregulation by DNA-damaging drugs in breast cancer cell lines.

## DISCUSSION

IGFBP-3 exerts both growth-stimulatory and apoptotic activity in breast cancer cells, and understanding how its expression is regulated by DNA-damaging agents has implications for predicting how IGFBP-3-expressing tumors may respond to chemotherapy [5]. In cancers where IGFBP-3 induces apoptosis, an increase in its expression in response to DNA damage would be clinically advantageous. Conversely, under conditions where IGFBP-3 is growth-stimulatory, downregulation of IGFBP-3 expression by chemotherapeutic drugs might contribute to their efficacy.

The factors that regulate the balance between growth stimulation and inhibition by tissue IGFBP-3 remain incompletely understood. There is some evidence that the “sphingolipid rheostat” [42] - a balance between pro-apoptotic lipids such as ceramides and pro-survival lipids such as sphingosine-1-phosphate - may be involved [5]. For example, exogenous IGFBP-3, which can enhance the apoptotic effect of ceramide [7], is reported to increase ceramide levels in the presence of doxorubicin [43]. Our observation that downregulating endogenous IGFBP-3 slightly decreased doxorubicin-induced caspase activation is consistent with this pro-apoptotic effect, although the contribution to apoptosis does not appear strong under these experimental conditions. Conversely, under conditions of cell survival (e.g. an absence of apoptotic stimuli), activation of the EGF receptor, a pro-survival tyrosine kinase, is potentiated by endogenous IGFBP-3 in several triple-negative breast cancer cell lines as well as MCF-10A, an effect requiring sphingosine kinase 1 and mediated by sphingosine-1-phosphate [16, 44]. Ceramide levels are expected to decrease under these conditions [43], possibly in association with a decrease in sphingomyelinases [45].

This study has shown for the first time that IGFBP-3 is regulated differentially in response to DNA damaging agents in different breast cell lines. Among the seven breast cell lines examined, the IGFBP-3 response to DNA damage may be divided into three groups: (i) ER-negative cells in which IGFBP-3 is expressed at moderate levels and is upregulated following DNA damage (MCF-10A and Hs578T), (ii) ER-positive cells in which IGFBP-3 is expressed at low levels and is also upregulated following DNA damage (MCF-7 and T47D), and (iii) ER-negative cells in which IGFBP-3 is expressed at high levels but is downregulated following DNA damage (MDA-MB-231, MDA-MB-436 and MDA-MB-468). Importantly, the three breast cancer cell groups described here may be reflected clinically in different types of response to chemo- or radiotherapy, and our findings may contribute to a more comprehensive characterization of their responsiveness in order to assign the most appropriate therapy to each case. Our data show that the topoisomerase II inhibitors doxorubicin and etoposide may cause downregulation of IGFBP-3 but the effects of other drugs used or proposed as treatments for TNBC, such as platinum-based antineoplastic agents including cisplatin and carboplatin [46] and PARP inhibitors such as olaparib and veliparib [47] on IGFBP-3 expression are not known.

Our findings suggest that the apoptotic response to DNA damaging therapy may be linked to the IGFBP-3 response. Hs578T cells, that responded to doxorubicin treatment with increased apoptosis, also induced IGFBP-3 mRNA in response to the drug, while MDA-MB-468 cells, with a much weaker apoptotic response, downregulated IGFBP-3 mRNA and protein in response to doxorubicin. Similarly, a previous study has contrasted the strong apoptotic response to doxorubicin in MCF-10A cells (in which IGFBP-3 is induced, Figure 2a) to the marked doxorubicin-resistance of MDA-MB-231 cells (in which IGFBP-3 is suppressed, Figure 2a) [48]. We showed recently that in MDA-MB-468 cells, endogenous IGFBP-3 may be involved in the early repair response to DNA damage, because siRNA-mediated knockdown of IGFBP-3 inhibited etoposide-induced activation of DNA double-strand break repair by non-homologous end-joining [18]. This effect was observed 4-6 h after treatment with etoposide while the proapoptotic effects of IGFBP-3 observed in this study were over 24-48 h of treatment. This suggests that the dual functions of IGFBP-3 in cell survival and death may in part be dependent on time, as well as on the level of DNA damage sustained by the cell. As noted above, the balance between pro-apoptotic and pro-survival sphingolipids may also be an important factor, since activation of sphingosine kinase 1 by IGFBP-3 [44] may generate sphingosine-1-phosphate, with potential effects on both cell survival and chemoresistance [49]. Measurement of tissue IGFBP-3 may have utility as a predictive marker for the response to DNA damaging therapy, since tumors with high IGFBP-3 expression before treatment may show greater resistance to the treatment than those that have relatively lower levels. This may be in part due to, or reflected by, DNA damage-induced downregulation of proapoptotic IGFBP-3 in certain breast cancers.

The p53 tumor suppressor protein [50] acts as a transcription factor to activate genes involved in cell cycle control and DNA damage repair; loss of p53 leads to genome instability and oncogenesis. Its expression and activation are induced by various stimuli, including DNA damage [51]. P53 activation-inactivation in response to stress depends on a repertoire of posttranslational modifications and interactions with proteins that induce p53 stabilization and subcellular relocalization, allowing it to induce appropriate sets of genes [51]. Although *IGFBP3* has been extensively documented as a p53-inducible gene [22, 23], it is not known how p53 mutational status influences IGFBP-3 expression in response to DNA damaging agents. We found that IGFBP-3 was upregulated by doxorubicin and etoposide in two cell lines with wild-type p53 (MCF-10A and MCF-7) and two with p53 mutations (Hs578T and T47D). The p53 mutants in Hs578T (V157F) and T47D (L194F) have previously been described as non-functional (IARC TP53 database), suggesting that doxorubicin-induced upregulation of IGFBP-3 in these cells may be p53-independent. However IGFBP-3 was downregulated following treatment with DNA-damaging agents in a subset of breast cancer cells that also contain p53 mutations: MDA-MB-231 (R280K), MDA-MB-436 (E204Fs) and MDA-MB-468 (R273H). These mutations all occur in the DNA binding domain of p53, altering its ability to bind to p53 response elements and induce transcription of many proteins [27, 52], possibly including IGFBP-3.

It is well-documented that certain mutant forms of p53 exert effects beyond those resulting from the loss of wild-type p53. Notably, many genes that are up or downregulated by wild-type p53 protein have been shown to be regulated in the opposite manner by particular mutants of p53 that display gain-of-function properties. The gain-of-function phenomenon was investigated as a possible explanation for the DNA damage-induced downregulation of IGFBP-3 observed in this study. Since exogenous IGFBP-3 can potentiate apoptosis following DNA damage [6–8, 29, 30, 53], the suppression of endogenous IGFBP-3 by an oncogenic mutant p53 might be expected to promote cancer cell survival. Unexpectedly, siRNA-mediated knockdown of mutant p53 by >80% had no effect on the downregulation of IGFBP-3 induced by doxorubicin or etoposide in MDA-MB-468 cells. This suggests that these DNA damaging agents inhibit IGFBP-3 expression by p53-independent mechanisms that are currently unknown.

Silencing of endogenous mutant p53 increased IGFBP-3 mRNA in MDA-MB-468 cells in the absence of DNA damaging agents. This finding extends the observations of Vikhanskaya *et al* who showed that *IGFBP3* promoter activity is suppressed by the R273H p53 mutant [54]. This p53 mutation, which is present in MDA-MB-468 cells, is known to abolish the sequence-specific DNA binding capabilities of p53 and thereby change its transcriptional activity [52]. Apart from this, however, it was not possible to show that the mutant form of p53 in MDA-MB-468 cells had gain-of-function properties because using either a p53-reactivating drug (PRIMA-1) or an siRNA approach, other differences in phenotype associated with mutant p53 function, such as reversal of p21 suppression, were not observed.

Owing to its role in facilitating carcinogenesis, mutant p53 and components of the p53 pathway are considered excellent candidates for targeted cancer therapies [55]. Direct targeting of mutant p53 may be possible with drugs that are designed to restore wild type function to mutant p53 [56], including PRIMA-1 [39], which is thought to reactivate mutant p53 by preventing its aggregation and encouraging correct folding, thereby promoting wild type-like function [56, 57]. Despite little evidence that PRIMA-1 behaved in our cell lines as reported in other studies, we tested the potential for this drug to reverse DNA damage-induced downregulation of IGFBP-3 levels in MDA-MB-468 cells. Increasing doses of PRIMA-1, alone and in the presence of doxorubicin, increased apoptosis (as measured by PARP cleavage) but had little effect on recognized p53 targets, such as p21 or IGFBP-3. In other studies, PRIMA-1 has been shown to reverse downregulation of mutant p53 targets, such as Apaf-1, PUMA, p21, Bax and Mdm2 [58–60]. Some of these effects may be transcription-independent [61, 62], which may explain why in the present study it induced apoptosis without affecting p21 or IGFBP-3. Collectively our data suggest that PRIMA-1 is not useful in reversing downregulation of IGFBP-3 induced by DNA-damaging agents in order to restore an IGFBP-3-induced apoptotic response of cancer cells to these drugs.

Taken together, our data suggest that DNA-damaging agents do not always regulate IGFBP-3 expression through p53, raising questions about other factors that may be involved. Various isoforms of other p53 family gene products, p63 and p73, may play a part as these proteins are generated by different promoters and alternative splicing but contain transactivation domains that can also activate p53 target genes [63]. Different isoforms of p63/p73 display different activities with full-length TA (transactivating) isoforms having tumor-suppressing properties, and ΔN isoforms, which lack the N-terminus, displaying oncogenic properties [40]. ΔNp63α has been shown to be upregulated by doxorubicin [64], and transcriptionally downregulates IGFBP-3 [41]. Thus it is possible that some cell lines that express ΔNp63α downregulate IGFBP-3 in response to doxorubicin. MDA-MB-468 cells are reported to express ΔNp63α [65], but we were unable to detect it either in this cell line or in MDA-MB-231 or -436 cells; therefore there is no direct evidence to support this explanation. The p73 isoform ΔNp73α also inhibits *IGFBP3* upstream promoter activity below basal levels [66] so this might also contribute to the IGFBP-3 downregulation response to DNA damaging drugs. Both mutant p53 and ΔNp73α have been shown to induce ΔNp63α in response to doxorubicin [67], linking the possible roles of p63 and p73 isoforms in this phenomenon.

In conclusion, we have shown that IGFBP-3 expression is differentially regulated by DNA-damaging agents in various breast cell lines. Despite its downregulation in cells that contain the R273H mutant form of p53, suggesting a possible p53 gain-of-function, this downregulation occurred even when p53 was silenced suggesting p53-independence. However, the subset of breast cancer cell lines in which IGFBP-3 was downregulated could be grouped by the level of IGFBP-3 expressed prior to treatment, with a decrease in IGFBP-3 expression in response to etoposide or doxorubicin occurring in those lines with the highest basal level of IGFBP-3 expression. IGFBP-3 may therefore provide an additional prognostic marker for response to DNA-damaging chemotherapy in some TNBC patients. The downregulation of IGFBP-3 that we have seen in this study may contribute to the resistance of some TNBC tumors to current treatments. We have thus proposed a potential new marker for improving the characterization of aggressive breast cancers.

## MATERIALS AND METHODS

### Reagents

Tissue culture plasticware was from Nunc (Roskilde, Denmark). Bovine serum albumin (BSA), epidermal growth factor (EGF), hydrocortisone and bovine insulin were from Sigma-Aldrich (St Louis, MO, USA). *Cholera* enterotoxin was from List Biologicals (Campbell, CA, USA). Fetal bovine serum (FBS) was from AusGeneX (Oxenford, QLD, Australia). RPMI 1640 medium was purchased from Thermo Scientific (Rockford, IL). DMEM/F12 medium, horse serum and trypsin/EDTA were from Gibco/Life Technologies (Mulgrave, VIC, Australia). Antibodies against PARP, total p53 and phospho-p53 (Ser-15) were purchased from Cell Signaling Technology (Beverly, MA, USA), while the antibody against p21 was from Merck Millipore (Billerica, MA, USA). Antibodies R30 and R100 against full-length human IGFBP-3 were generated in-house [68]. The β-actin antibody was from Sigma-Aldrich. Molecular weight markers PageRuler (Fermentas Life Sciences, Burlington, ON, Canada) or Himark (Invitrogen, Carlsbad, CA) were used for Western blot analysis.

### Tissue culture

Breast cancer cell lines were purchased from the American Type Culture Collection (Manassas, VA, USA) and maintained in RPMI 1640 medium supplemented with 5% FBS and 10 μg/ml bovine insulin. Hs578T, MDA-MB-231, MDA-MB-436 and MDA-MB-468 cell lines are all triple-negative and express moderate to high levels of IGFBP-3, while MCF-7 and T47D cell lines are ER-positive and express very low levels of IGFBP-3 (Supplemental Table 1). The phenotypically normal MCF-10A breast cell line (also ER-negative) was a gift from Drs Robert Pauley and Herbert Soule from the Karmanos Cancer Institute (Detroit, MI, USA). MCF-10A cells were maintained in DMEM/F12 (1:1) medium supplemented with 5% horse serum, 10 μg/ml insulin, 10 ng/ml EGF, 0.5 μg/ml hydrocortisone and 100 ng/ml *Cholera* toxin. Cell lines were maintained continuously for a maximum of 40 passages before fresh cultures were established from frozen stocks. The Hs578T, MDA-MB-231 and MCF-10A lines were authenticated by cell typing using short tandem repeat DNA profiling at CellBank Australia (Westmead, NSW, Australia). Cryopreserved stocks of the other cell lines were established within 1 month of receipt from ATCC, and fresh cultures for use in experiments were established from these stocks. Conditioned media were tested for mycoplasma infection on a monthly basis (MycoAlert Mycoplasma Detection Kit, Lonza).

### Cell treatments

Cells were seeded at 2×10^5^ cells/well (MCF-10A and Hs578T) or 3×10^5^ cells/well (MCF-7, T47D, MDA-MB-231, MDA-MB-436 and MDA-MB-468) into 6-well culture plates or half that cell number/well in 12-well culture plates, and allowed to grow for 24 h before washing with phosphate buffered saline (PBS) and replacing with medium containing DNA-damaging drugs (1 μM doxorubicin or 20 μM etoposide) or 25-50 μM PRIMA-1 (all from Sigma-Aldrich). Where necessary, DMSO was used as a vehicle control for untreated cells. Cells were returned to the incubator for 24-48 h before harvesting for analysis of various endpoints.

### siRNA-mediated gene silencing

Cells were grown in flasks to approximately 80% confluency, harvested and pelleted by centrifugation at 1300 rpm for 5 min. The medium was aspirated and the cells resuspended in the appropriate transfection solution (Kit T for Hs578T, and Kit V for MCF-7 and MDA-MB-468) (Lonza, Mt Waverley, VIC, Australia) before transfecting with 200 pmol AllStar negative control, IGFBP-3 or p53 siRNA (Qiagen FlexiTube siRNA) using the Amaxa Electroporation System (Lonza) and recommended transfection programs (A-023 for Hs578T, P-020 for MCF-7 and X-005 for MDA-MB-468). The electroporated cell suspension was immediately diluted into fresh medium (37°C) before seeding into 6- or 12-well culture plates. Cells were then incubated overnight before treatment for 4, 24 or 48 h, as indicated for individual experiments. Cell lysates from replicate wells were collected 24 h post-transfection and used to validate knockdown efficiency by qRT-PCR or immunoblotting methods as described below.

### Quantitative real-time PCR (qRT-PCR)

Cells were seeded in 6-well plates and treated as indicated for individual experiments before removing the medium, washing in ice-cold PBS and lysing directly in the well with 0.5 ml TRIzol Reagent (Invitrogen). RNA was isolated from the TRIzol lysate according to manufacturer’s instructions. The final RNA pellet was resuspended in RNase-free water and stored at −80°C. First-strand cDNA was synthesized using 1 μg total RNA with the SuperScript III First-Strand Synthesis SuperMix kit (Invitrogen) as per manufacturer’s instructions. The cDNA samples were diluted 1/4 in RNase-free water (Qiagen) before combining with master mixes containing TaqMan Universal PCR Master Mix, No AmpErase UNG (Life Technologies, Mulgrave, VIC, Australia) and probes for genes of interest (*IGFBP3*, *TP53* or *CDKN1A*) or the housekeeping gene (*GAPDH*) (TaqMan Gene Expression Assays, Life Technologies). The qRT-PCR was performed in triplicate for each sample using a Rotor-Gene 3000 rotary-based thermocycler (Corbett Life Science, Mortlake, NSW, Australia) or an AB7900 instrument (Applied Biosystems, Life Technologies). Data were acquired using the operating software supplied with the machines. Quantities of the genes of interest were calculated relative to GAPDH and normalised to one control/untreated sample replicate.

### SDS-PAGE and Western blotting

Lysates from treated cells were prepared for Western analysis as follows. Conditioned medium was collected and centrifuged to pellet detached cells. Adherent cells were washed with ice-cold PBS, lysed in Laemmli sample buffer (62.5 mM Tris, 20 g/L SDS, 10% (v/v) glycerol, 50 mM dithiothreitol (DTT), 0.1 g/L bromphenol blue, pH 6.8) directly in the culture wells with gentle shaking for 15 min at 4°C and pooled with cells recovered from conditioned medium. Cell lysates were heat-denatured at 95°C for 5 min, and equal volumes of each sample were loaded onto gels for separation by SDS-PAGE, then transferred to Hybond-C nitrocellulose membrane (Amersham Biosciences). Membranes were washed briefly in Tris-buffered saline (TBS-T: 10 mM Tris, 0.15 M NaCl, 0.1% (v/v) Tween-20, pH 7.4) and blocked with 5% low-fat milk/TBS-T and incubated with primary antibody diluted in 1% BSA/TBS-T overnight at 4°C. Membranes were washed, then incubated with the appropriate secondary antibody horseradish peroxidase conjugates (GE Healthcare, Rydalmere, NSW, Australia). Immunoreactive bands were visualized using SuperSignal West Pico Chemiluminescent substrate (Pierce, Rockford, IL, USA) and a LAS-3000 imaging system and ImageReader LAS-3000 Lite software (FujiFilm, Tokyo, Japan). MultiGauge image software (FujiFilm) was used for densitometry of bands. Membranes were re-probed with β-actin antibody as loading control after visualization of the protein of interest.

### Caspase-3 activity assay

Cells were seeded in 6-well plates and incubated overnight before treating with doxorubicin or etoposide for 24 or 48 h. Medium containing detached cells was collected, centrifuged at 4000 rpm for 5 min, and the supernatant stored for analysis of IGFBP-3 by SDS-PAGE. The cell pellet was pooled with the lysates prepared from attached cells which had been washed with cold PBS before lysing with cell lysis buffer (10 mM Tris, 100 mM NaCl, 1 mM Na_2_EDTA, 0.01% (v/v) Triton X-100, pH 7.5) for 20 min at 4°C with shaking. Samples were frozen at −80°C overnight, then thawed at 37°C, immediately placed on ice for 15 min, then vortexed and centrifuged at 4000 rpm for 10 min at 4°C. The supernatant was transferred to a fresh 1.5 ml Eppendorf tube and the pelleted cell debris discarded. Total protein in cell lysates was measured using the Protein Assay Dye Reagent Concentrate (Bio-Rad, Hercules, CA, USA) and the protein concentration of each sample adjusted to 200 μg/ml by diluting in cell lysis buffer. Assays were performed in 96 well, black-bottom plates A standard curve for quantification of caspase-3 activity was prepared using amino-4-methylcoumarin (AMC; Sigma-Aldrich) in working reaction buffer (20 mM PIPES, 4 mM Na_2_EDTA, 2 g/L CHAPS, 10 mM DTT) over the concentration range 1-100 mM. Substrate solution (reaction buffer containing 2 mM acetyl-Asp-Glu-Val-Asp-AMC (Enzo Life Sciences, Farmingdale, NY, USA) was prepared immediately before use and added to wells containing cell lysate or lysis buffer (as a blank) at a 1:1 ratio. Fluorescence (AMC excitation/emission = 342 nm/441 nm) was measured using a Victor3 1420 Multilabel Counter (PerkinElmer, Waltham, MA, USA) and associated Wallac1420 software. Nine readings at 20 min intervals were recorded for each assay. Fluorescence data were corrected for background fluorescence and the relative amount of caspase-3/7 activity was calculated as the difference between the corrected reading at 160 min and the corrected reading at 40 min, or appropriate time points at which a linear response was observed.

### Data analysis and statistics

Data were analysed using Prism version 5 (GraphPad Software, La Jolla, CA, USA). One-way or two-way analysis of variance (ANOVA) tests were used, followed by the Bonferroni’s Multiple Comparison post-hoc test. Experiments were carried out at least twice in duplicate or triplicate and *P* values <0.05 were considered significant.

## SUPPLEMENTARY MATERIAL FIGURES


