# Supplementary material for: Involvement of p53 in insulin-like growth factor binding protein-3 regulation in the breast cancer cell response to DNA damage
Source: Oncotarget. 2015 Sep 10;6(29):26583–98. doi: 10.18632/oncotarget.5612 (PMC4694938; doi:10.18632/oncotarget.5612)
Supplement: Supplementary file 1 [file oncotarget-06-26583-s001.pdf]

Involvement of p53 in insulin-like growth factor binding protein-3 regulation in the breast cancer cell response to DNA damage

Supplementary Material

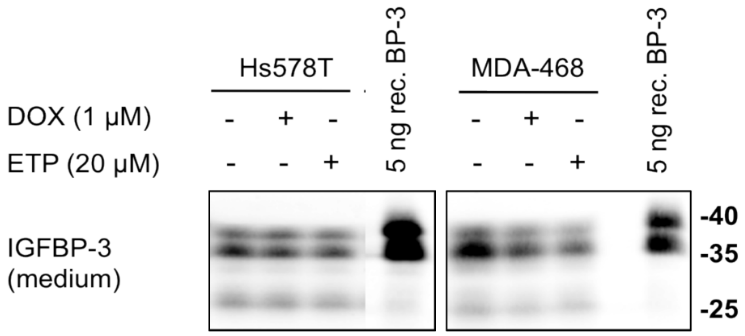

Figure S1. Regulation of secreted IGFBP-3 in Hs578T and MDA-MB-468 cells in response to DNA damaging drugs. Conditioned medium was collected from Hs578T and MDA-MB-468 cells following 24 h treatment with 1 μM doxorubicin (DOX) or 20 μM etoposide (ETP), and subjected to Western blotting for IGFBP-3, using 5 ng recombinant IGFBP-3 (rec. BP-3) as a standard. Note the band at ~27 kDa representing proteolysed IGFBP-3 in some samples. Molecular weight markers are shown on the right.

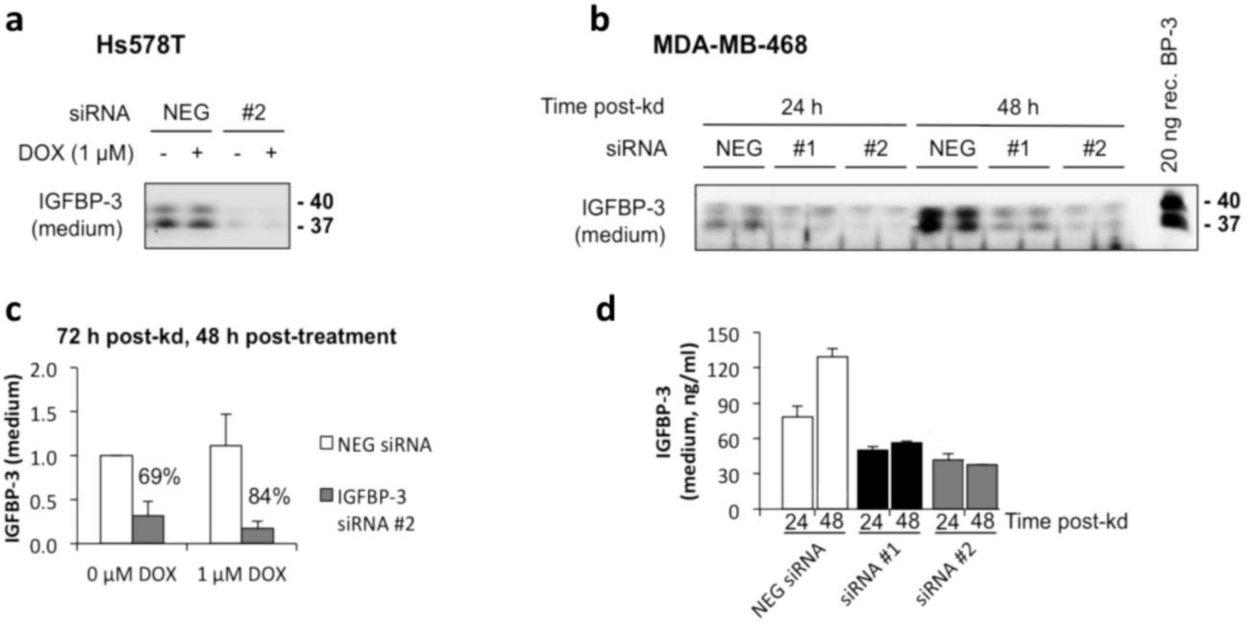

Figure S2. SiRNA-mediated knockdown of IGFBP-3. Conditioned medium was collected (a) 72 h post-knockdown (kd) (Hs578T) or (b) 24 h and 48 h post-kd (MDA-MB-468). IGFBP-3 protein (37 and 40 kDa doublet) was detected in the medium by Western blot. Signal intensities were measured by densitometry and plotted (c) relative to the untreated NEG siRNA control, with percentage knockdown indicated (Hs578T) or (d) as a concentration relative to a 20 ng recombinant IGFBP-3 standard (rec. BP-3) (MDA-MB-468). Data are mean values ± SEM of at least two experiments in duplicate for each cell line.

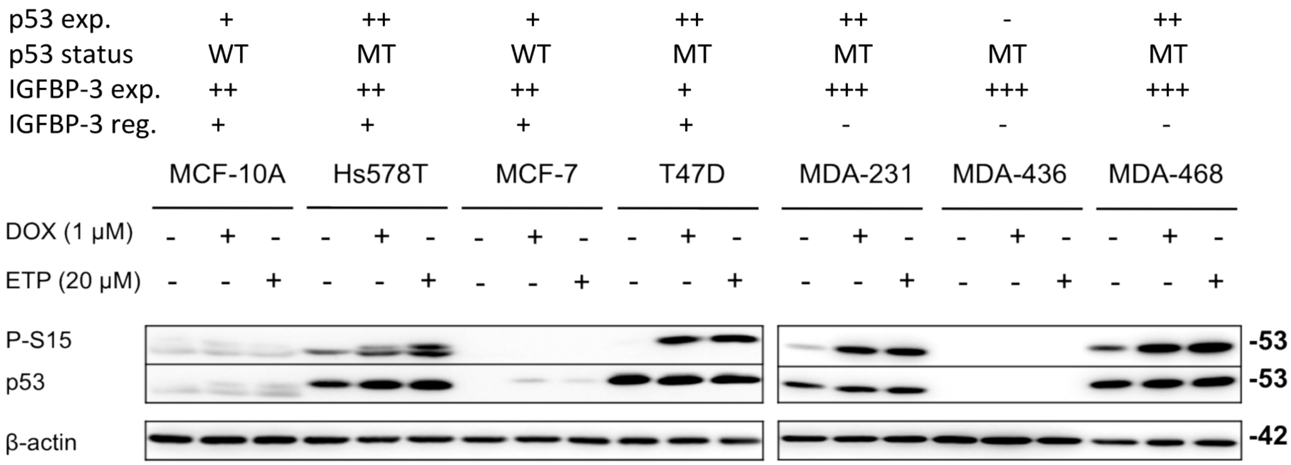

Figure S3. p53 expression in breast cell lines. Cells were harvested following 4 h treatment with 1 μM doxorubicin (DOX) or 20 μM etoposide (ETP). Lysates were subjected to Western blotting to detect phospho-p53 (P-S15) and total p53. β-actin is shown as a loading control. WT, wild type; MT, mutant.

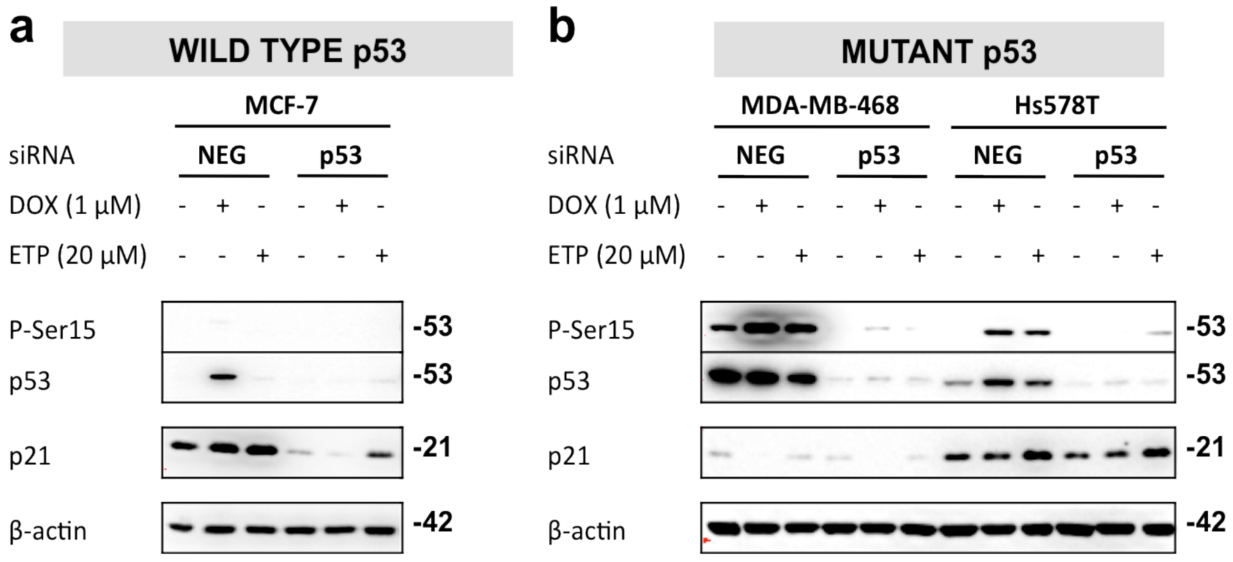

Figure S4. SiRNA-mediated knockdown of p53 and its effect on p21 protein expression. Cells were harvested 48 h after p53 siRNA transfection and following 24 h treatment with 1 µM doxorubicin (DOX) or 20 µM etoposide (ETP). Lysates from (a) wild type p53-containing cells (MCF-7) and (b) mutant p53-containing cells (MDA-MB-468 and Hs578T) were subjected to Western blotting to detect phospho-p53 (P-Ser15), total p53, total p21 and β-actin. CTL, control untreated; NEG, negative siRNA.

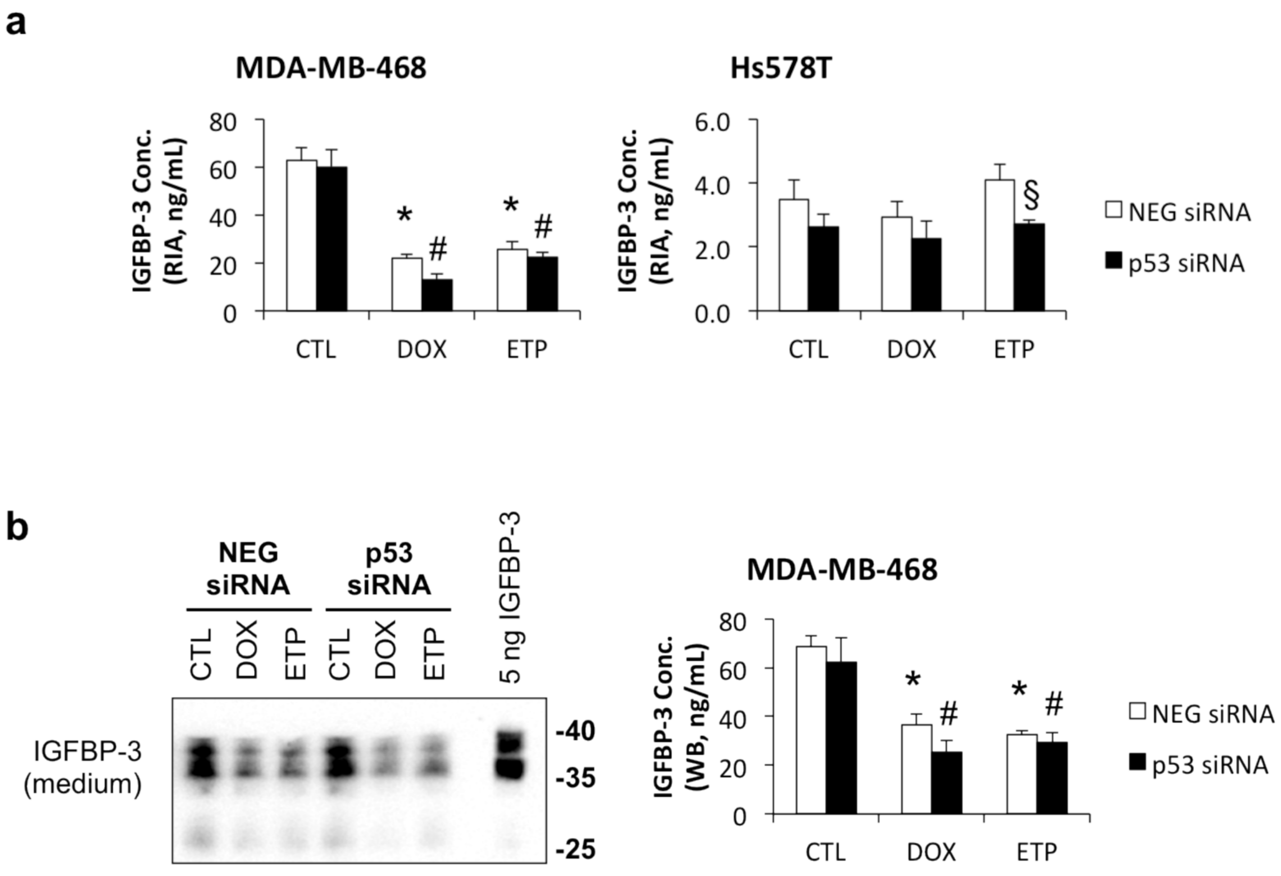

Figure S5. Effect of siRNA-mediated knockdown of p53 on IGFBP-3 protein expression. Secreted IGFBP-3 was measured in medium by (a) radioimmunoassay and (b) Western blot analysis. CTL, control untreated cells; DOX, doxorubicin; ETP, etoposide; NEG, negative siRNA.

*$P<0.05$ comparing NEG siRNA DOX/ETP to NEG siRNA CTL.

#$P<0.05$ comparing p53 siRNA DOX/ETP to p53 siRNA CTL.

§$P<0.05$ comparing p53 siRNA to NEG siRNA.

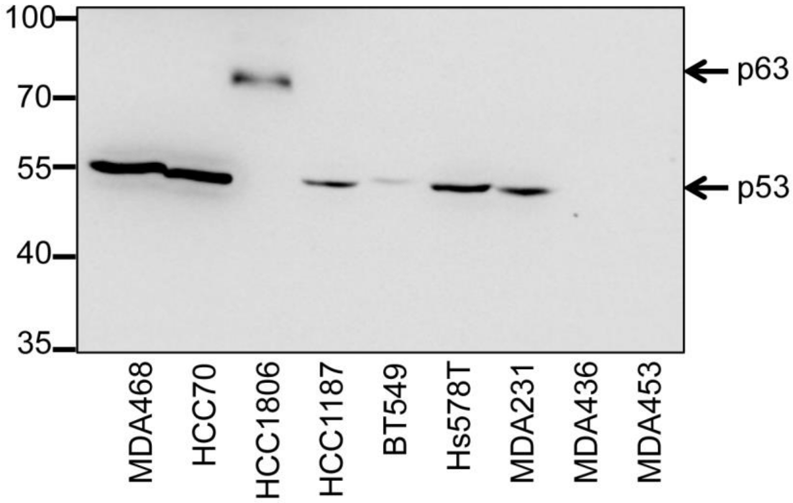

Figure S6. Detection of p63 protein in human breast cancer cell lines. The breast cancer cell lines MDA-MB-468, HCC70, HCC1806, HCC1187, BT549, Hs578T, MDA-MB-231, MDA-MB-436 and MDA-MB-453 were screened for expression of full-length (transactivating) p63 (approx. 75 kDa) and its truncated isoform ΔNp63α (approx. 40 kDa) by Western blot analysis using an antibody that is reported to detect both forms of p63 (Cell Signaling Technologies; Beverly, MA, USA). No cell line had a detectable p63 isoform around 40 kDa. After probing with p63 antibody, the membrane was reprobed (without stripping) with antibody against p53. Cell lines with p53 insertion/deletion mutations (HCC1806, MDA-MB-436, MDA-MB-453) had no detectable p53. Location of molecular weight markers is indicated on the left.
